# Effect of Topically Administered Natural Compounds on Cesarean Section Healing—A Retrospective Observational Study

**DOI:** 10.3390/ph19091377

**Published:** 2026-08-31

**Authors:** Melinda Ildiko Mitranovici, Raluca Moraru, Septimiu Voidazan, Florin Buicu, Ion Petre, Liviu Moraru

**Affiliations:** 1 Department of Obstetrics and Gynecology, Emergency County Hospital Hunedoara, 14 Victoriei Street, 331057 Hunedoara, Romania; mitranovicimelinda@yahoo.ro; 2 Department of Anatomy, University of Medicine, Pharmacy, Science and Technology “George Emil Palade” of Targu-Mures, 540139 Targu Mures, Romania; liviu.moraru@umfst.ro; 3 Department of Epidemiology, University of Medicine, Pharmacy, Sciences and Technology, “George Emil Palade”, 540142 Targu Mures, Romania; septimiu.voidazan@umfst.ro; 4 Department of Public Health and Management, University of Medicine, Pharmacy, Science and Technology “George Emil Palade” of Targu-Mures, 540139 Targu Mures, Romania; florin.buicu@umfst.ro; 5 Department of Functional Sciences, Medical Informatics and Biostatistics Discipline, “Victor Babes” University of Medicine and Pharmacy, 300041 Timisoara, Romania; petre.ion@umft.ro

**Keywords:** post-cesarean wound, Reparis, Cicatridine, natural compounds, topical gel

## Abstract

Natural compounds in topical gel formulations are recognized for their ability to enhance wound healing, particularly in the context of postoperative care, including that of cesarean sections. **Background**: Cesarean sections are among the most common surgical procedures globally, highlighting the importance of their successful healing. Complementary medicine aimed at pain relief and wound healing after a cesarean section has been investigated worldwide, and natural compounds are considered safe for topical use during pregnancy and breastfeeding. The aim of our study is to demonstrate the efficacy of the use of natural compounds in post-cesarean scar treatment. **Materials and Methods**: We compared Reparis, a topical gel entirely composed of natural compounds such as *Centella asiatica* and *Aloe vera*, with Cicatridine, a topical gel containing synthetic and natural compounds, such as almond oil, in post-cesarean wound treatment. We conducted an observational retrospective study in County Hospital Hunedoara between 26 February and 31 May 2026, evaluating specific measures that relate to the cosmetic results of scars after surgical procedures in our unit, with 50 patients using Reparis twice a day compared to the control group, which itself consisted of 50 patients using Cicatridine twice a day for 30 days. We employed the POSAS, an internationally validated questionnaire for evaluating patient and surgeon opinions regarding the appearance of a scar. **Results**: The most important difference favoring Reparis was observed after 6 months (*p* = 0.0001). In addition, Cicatridine exhibits a positive correlation with BMI; Reparis can be used in cases of higher BMI with good healing results. **Conclusions**: Natural compounds alone, such as Reparis, pose promising potential for wound healing, based on the findings of our observational study. The therapeutic properties of traditional medical plants have been verified but not always in controlled clinical trials. Our findings still require external validation.

## 1. Introduction

*Centella asiatica* and *Aloe vera* are recognized for their stretch-mark-reducing properties [1] and are also employed in wound repair [2]. Natural compounds in topical gel formulations are recognized for their ability to enhance wound healing, particularly in the context of postoperative care, including that of cesarean sections. They are used to improve skin elasticity and are considered safe for topical use during pregnancy and breastfeeding [2].

Wound healing involves four stages of tissue repair: hemostasis, inflammation, proliferation, and remodeling [2,3]. In the first stage, bleeding is followed by vasoconstriction, with platelets being the essential cells involved, while fibrinogen is the crucial matrix component. During hemostasis, a thrombus is formed by the platelet plug and fibrin mesh [2]. The thrombus releases growth factors; wound-healing cells adhere to the endothelium and inflammation occurs, controlling bleeding and preventing infection. Monocytes, neutrophils, mast cells, and lymphocytes migrate to the wound together with platelets and produce Tumor Necrosis Factor alpha, cytokines, enzymes, and platelet-derived growth factor [2]. This process may cause swelling, discomfort, and redness [1,2]. In the third phase, new granulation tissue grows via proliferation and extracellular matrix (ECM) deposition. The fourth phase is remodeling, characterized by neovasculature regression and scar tissue rebuilding with ECM deposition; type I collagen replaces the weaker type III collagen fiber [2]. Apoptosis is employed to eliminate redundant cells. Collagen crosslinks, strengthening the wound and thinning the scar. Several factors, such as infection, diabetes, vascular deficiency, or metabolic disorders, may interfere with the process [2,4].

Healing may be improved by maintaining a clean wound environment and ensuring that it is free of bacteria. Standard wound management involves the removal of tissue debris, maintaining wound hygiene, and applying a wound dressing, thus preventing infections [2]. Ideal wound dressings should relieve pain, induce hemostasis, attach to healthy skin, prevent infection, and accelerate the healing process, all while remaining non-toxic [2,4]. Hydrogels represent the preferred type of wound dressing. Antiseptic and antimicrobial agents have long been used topically to treat wounds and impart proven benefits [2,4]. Growth factors are being explored topically as a form of wound treatment, showing significant impacts on skin healing [5]. For topical use, vitamin A is often combined with vitamin E, thus imparting antioxidant effects, enhancing elasticity, and reducing skin inflammation. This combination has the potential to normalize keloid fibroblasts, preventing hyperproliferation [6]. Dexpanthenol, a component of vitamin B, facilitates wound healing, acting as an anti-inflammatory [7]. The ECM is a network of macromolecules that is essential for coordinating the wound-healing process. The ECM is abundant in collagen, which offers structural stability. Elastin is another component that contributes to elasticity, while laminin, fibronectin, and vitronectin play important roles in cell adhesion and migration. The extracellular matrix is essential for sustaining stem cell environments and their capacity for regeneration [8].

Cesarean sections are among the most common surgical procedures globally, making the successful healing of the residual wound particularly important [9]. Complementary medicine for pain relief and wound healing after a cesarean section has been investigated worldwide [9]. Natural compounds are viable alternatives that possess multitarget bioactivity, enhancing the regenerative microenvironment. They can reduce oxidative stress, influence collagen synthesis, and regulate angiogenesis [8]. In wound therapy, natural compounds such as rosemary oil, *Aloe vera*, *Centella asiatica*, and curcumin have been employed due to their antibacterial, angiogenic, and regenerative effects [2,9,10,11,12,13,14,15]. According to Oryan et al., topical treatment with *Aloe vera* improved the morphological properties of skin lesions in animals [2,11].

Further translational research is required to determine how modulation of these processes may reduce scarring and enable clinically meaningful outcomes [8].

Scar treatment should be implemented in a planned manner, using appropriate pharmacological agents. The efficacy of many drugs used for this purpose has yet to be investigated [12].

While patients place great importance not only on their health but also on their esthetic appearance, given the impact on women’s mental health during the sensitive postpartum period, there is a significant need for effective treatment for cesarean scars. Natural products considered during breastfeeding are gaining ground in the management of the postpartum period.

The aim of our study is to compare Reparis, a topical gel composed exclusively of natural compounds such as *Centella asiatica* and *Aloe vera*, with Cicatridine, a topical gel containing synthetic and natural compounds, such as almond oil, in post-cesarean wound treatment. The purpose of this study is to evaluate the efficacy of natural compounds for use in treating post-cesarean scars.

## 2. Results

Based on clinical patient characteristics such as age, body mass index (BMI), parity, associated pathologies, laboratory analyses, complications during and after surgery, and length of hospitalization (in days), no significant differences were observed (Table 1).

### 2.1. Results After 1 Week

All characteristics are presented in centralized tables, and all statistically significant values are highlighted in bold for greater visibility. For the “color” parameter, the *p*-value was 0.0001 because, on a 10-point Likert scale, a score of 1 appeared more frequently in the Cicatridine group (range 1) than in the Reparis group (range 6). The same pattern was observed for “stiffness” and “thickness.” When the items were summed, a significant difference was identified one week postoperatively (*p* = 0.003), with a range of 3 for Cicatridine and a range of 37 for Reparis (Table 2). Although no significant difference was observed for “itchiness” between the two products, pain was significantly reduced in the Cicatridine group 1 week after treatment.

### 2.2. Results After 6 Weeks

No significant differences were recorded between Reparis and Cicatridine use after 6 weeks (Table 3).

### 2.3. Results After 6 Months

At 6 months, the pattern was reversed, with a score of 1 appearing more frequently in the Reparis group than in the Cicatridine group in terms of color, stiffness, thickness, and irregularity on the 10-point Likert scale. In the Cicatridine group, scores > 2 were more frequently observed. Furthermore, when individual item scores were summed, a total score > 7 occurred more frequently in the Cicatridine group (*p* = 0.0001), suggesting that Reparis possesses a greater capacity to promote cesarean scar healing (Table 4).

Pain and itchiness were not significantly alleviated by Reparis compared with Cicatridine at either 6 weeks or 6 months; however, statistically significant differences were observed in color, stiffness, thickness, and irregularities, with Reparis being superior in terms of long-term wound healing.

### 2.4. Correlation with Different Parameters

Non-parametric Spearman correlations were performed. An asterisk * indicates statistical significance.

The correlations between the variables of interest in the Reparis group are presented in Table 5, enabling a detailed interpretation of selected associations. The POSAS parameter scores were summed to obtain a total score (t) at 1 week (w), 6 weeks, and 6 months. For example, between t/1 w, t/6 w, t/6 m, and length of hospitalization, significant positive correlations were observed: as the length of hospital stay increased, the total POSAS score also increased, frequently exceeding the threshold of 6 (Table 5).

The same approach was applied to the Cicatridine group. Several significant correlations were observed, with positive correlations indicating that both variables increased in tandem. As described above, longer hospitalization was positively correlated with total POSAS score, with higher scores frequently exceeding 6. Similarly, higher BMI was positively correlated with the total POSAS score (Table 6).

In the Reparis group, the total POSAS score exhibited a positive correlation with the length of hospitalization, whereas in the Cicatridina group, positive correlations were observed with BMI and age. These findings suggest that wound healing is impacted by longer hospitalization due to a range of factors, in addition to BMI, even when Cicatridine is used to repair the lesion. However, in the case of Reparis, BMI does not appear to contribute to more effective wound healing.

## 3. Discussion

In our study, the assessed natural compounds are demonstrably efficacious, possessing regenerative and antibacterial effects. After one week of Cicatridine use, significant differences were observed in the summed item scores one week postoperatively (*p* = 0.003), with a range of 3 for Cicatridine and a range of 37 for Reparis (Table 2).

After 6 weeks, no significant differences were observed. However, after 6 months, higher scores were observed for Cicatridine use in terms of color, stiffness, thickness, and irregularities; furthermore, by summing the items, a significantly higher score (*p* = 0.0001) was obtained for Cicatridine use, indicating that Reparis provides significantly better wound-repair properties, likely due to its mechanism of action. While Cicatridine is beneficial in soothing inflammation and providing wound hydration, Reparis is also involved in tissue regeneration, collagen stimulation, and angiogenesis [13,14]. Applying Pearson’s correlation, a positive correlation was found between the use of both Reparis and Cicatridine and the number of days of hospitalization. In addition, Cicatridine exhibits a positive correlation with BMI; Reparis can be used in cases of higher BMI with satisfactory healing results. The POSAS is the only scale that considers subjective symptoms of pain and pruritus, yet it does not quantify whether these factors interfere with quality of life, representing a key limitation; however, in long-term assessments, no significant differences were found [16].

Recent evidence indicates that ROS significantly influence wound healing and skin regeneration by regulating inflammation, cell proliferation, angiogenesis, and extracellular matrix formation [8]. Natural compounds in topical use have been investigated, with Haq et al. reporting a significant difference (*p* = 0.001) in favor of *Centella asiatica* gel compared with the *Aloe vera* group [1]. In their study, Puttarak observed intensive pharmacological effects in wound healing using a combination of polysaccharides from *Aloe vera* gel and pentacyclic triterpenoids, abundant in *Centella asiatica* extracts [17]. In the same manner, in our study, Reparis containing *Centella asiatica* and *Aloe vera* demonstrated a significantly stronger wound-healing capacity than Cicatridine.

A total of 26 published clinical trials highlight herbal wound-healing formulations with significant efficacy [15].

Honey is a natural compound with antimicrobial and anti-inflammatory properties that promote tissue regeneration and scar reduction [16,18]. Curcumin’s wound-healing potential is due to its anti-inflammatory, anti-infectious, collagen deposition, and antioxidant activities [19,20]. Quercetin found in *Ginkgo biloba* exhibits antioxidant properties. *Centella asiatica*, or Asiatic pennywort, has a long history of use, imparting anti-inflammatory and antibacterial properties in wound healing while also promoting angiogenesis [21]. The medicinal plant *Centella asiatica* contains Asiaticoside, promoting fibroblast proliferation and activation of specific growth factors with significant healing properties [19,21]. Medicinal plants possess demonstrated potential for use in wound management [6]. Identical results were obtained with Reparis, which contains *Centella asiatica,* in our study, compared with the chlorhexidine contained in Cicatridine.

Researchers observed a decrease in tissue damage associated with the recruitment of myofibroblasts during the proliferative stage of wound healing [22]. Wound-healing technologies are employed to incorporate herbal bioactives into biopolymeric wound dressings [23]. These dressings incorporate natural polymers, such as hyaluronic acid, chitosan, gelatin, and pectin, actively involved in cell adhesion and proliferation, which are necessary for wound repair. *Curcumin*, quercetin, *Aloe vera*, and *Centella asiatica* are among the herbs included in biopolymeric formulations with anti-inflammatory, antioxidant, and antibacterial effects [23]. In the same manner, Reparis and Cicatridine both contain hyaluronic acid as a biopolymer.

Wound dressings based on various natural polymers, for example, polysaccharides (chitosan, heparin, alginates, and chondroitin) or proteins (collagen, fibrin, and keratin), are used for wound management because they provide an optimal microenvironment for cell proliferation, migration, and differentiation, also finding use in regenerative medicine [24].

Despite advancements in trauma management, infection remains a substantial cause of death among millions of individuals with wounds worldwide. Natural remedies derived from plants are justified by their therapeutic properties [25]. In this regard, hydrogel-based systems are ideal among all drug-delivery systems due to their controlled release properties and biocompatibility [25]. During the healing process, platelet aggregation in the hemostasis stage is followed by the arrival of neutrophils and macrophages, which are involved in pathogen defense and tissue repair [22,25]. Angiogenesis and epithelialization are post-inflammatory phases. In addition, fibroplasia involves the proliferation of fibroblasts and the production of extracellular matrix (ECM), which provides structural support [25]. Collagen fibers are reorganized along lines of tension, and extra cells undergo apoptosis [25,26]. Hydrogel-based drug-delivery systems offer excellent biocompatibility and can encapsulate herbal compounds for delivery to the wound site while also enhancing their therapeutic potential [27]. While Cicatridine contains chlorhexidine and imparts benefits due to its antimicrobial role, Reparis contains natural compounds with significant efficacy, with a lower POSAS score than Cicatridine, resulting in stronger wound-healing capacity.

Keloid formation can present various challenges; however, it can be prevented by natural compounds such as *Centella asiatica* to improve collagen formation [28]. These natural materials are less expensive than advanced treatments due to their wide availability [25]. *Centtella asiatica* was used in the Reparis formulation in this study.

From herbal applications in ancient cultures to the development of bioengineered dressings in contemporary medicine, the field of wound healing reflects a continuous trajectory of innovation and adaptation [4]. *Aloe vera* is widely used due to its antioxidant capacity, antimicrobial activity, and its involvement in fibroblast proliferation and collagen synthesis. Many wound dressings and emulsions contain this compound [4,29]. Herbal combinations constitute a multitargeted potential avenue in wound management [4]. Topical wound-healing polyherbal formulations have not yet been associated with any notable side effects, such as erythema, skin irritation, or edema, in animal models. However, studies on the adverse effects of these compounds are scarce. Plants are selected for combination through a process designed to avoid incompatibility [4]. In our study, only two patients exhibited skin irritation: one in the Reparis group and the other in the Cicatridina group.

Topical foams are now extensively used in dermatological therapies [30]. Almond is also used as an emollient in cosmetics. Herbal extracts used in combination with chlorhexidine can reduce scar and wrinkle formation [31]. No studies comparing these formulations with *Aloe vera* and flavonoids from *Calendula officinalis* or *Centella asiatica* were identified, as detailed in the present study. These natural compounds can be incorporated into collagen or chitosan matrices. In such combinations, they stimulate granulation, inhibit inflammation, and accelerate epithelialization [4]. Taheri et al. compared olive oil use with placebo after cesarean section among 34 women in the study group, 34 women in the placebo group, and 35 women in the control group. They found a statistically significant difference between the intervention group and placebo group, intervention and control groups, and placebo and control groups in terms of pain intensity (*p* < 0.05 in all three cases). They also found a statistically significant difference in terms of wound-healing scores (*p* < 0.05 in both cases) [32].

Multiple factors account for the increasing number of cesarean delivery wound complications in the United States, for example, obesity and diabetes mellitus, for which modern wound-healing products are sought [33]. Wound dressings based on multiple natural polymers, for example, polysaccharides (chitosan, heparin, alginates, chondroitin) or proteins (collagen, fibrin, keratin), are used in wound management because they provide an optimal microenvironment for cell proliferation, migration, and differentiation while also finding use in regenerative medicine [34]. *C. asiatica* has demonstrated wound-healing properties resulting from improved angiogenesis and a stimulating effect on Fibroblast Growth Factor (FGF) and Vascular Endothelial Growth Factor (VEGF) production. In addition, it exerts an anti-inflammatory effect by reducing the levels of Interleukin-1β (IL-1β), Interleukin-6 (IL-6), and Tumor Necrosis Factor α (TNFα). *C. asiatica* extract has also been traditionally used for the management of keloids [31]. In our study, Reparis, which contains C. asiatica, was found to be associated with fewer complications than Cicatridine in terms of increased BMI.

Stretch marks are common in postpartum women, and herbal treatments such as *Centella asiatica* and *Aloe vera* are recognized for their skin-regenerating properties. Haq, in a study involving 32 postpartum women, demonstrated that *Centella asiatica* gel is more effective than *Aloe vera* gel in reducing postpartum stretch marks and improving skin elasticity [1]. Research suggests that *Centella asiatica* may be more effective than *Aloe vera* alone in treating specific postpartum skin issues such as stretch marks. *Centella asiatica* has been used in traditional medicine systems; more recently, however, it has gained global recognition in skincare [35]. The key limitation of our study is that it is an observational, single-center, retrospective research work. However, to our knowledge, this is the first comparative study of its kind on Reparis and Cicatridine use. Our research requires external validation to confirm our findings. Another major limitation is that the investigated products have a complex multicomponent formulation, which is why the study design does not allow the individual contribution of each active ingredient to be determined.

The novelty of our study on such tissue-repairing products consists of the use of natural compounds in wound healing, which bring several components in a new combination that has not been investigated and compared yet. Reparis contains 12 different compounds made from 100% natural products, with a high regenerative profile. Both utilize biological structural matrices such as hyaluronic acid rather than conventional heavy grease or harsh antibiotic barriers. They also exhibit multipurpose adaptation, expanding the formulation from cutaneous ointments to specialized delivery formats such as gels, targeting distinct epithelial surfaces without systemic side effects, supporting natural tissue remodeling [36,37].

Recognizing the important role and clinical value of traditional medicine, we observed across several studies involving 36 plants that *Centella asiatica*, *Curcuma longa*, and *Aloe vera* are particularly prominent. Traditional practices still offer valuable lessons, including unexplored combinations that could have a place in the contemporary therapeutic inventory [3]. Active phytocompounds quickly stop bleeding, efficiently kill bacteria, and selectively induce fibroblast apoptosis or cell-cycle arrest in over-proliferating scar tissues while supporting healthy re-epithelialization. Natural compounds have a higher bioavailability, and accelerate collagen deposition and blood vessel formation, being safer, with lower-cost profiles and fewer adverse dermatological side effects [3,36,37].

While in vitro studies show basic molecular mechanisms and lack a full immune system or organ network, animal models show how the treatment acts in a living body, but we have to state clearly that animal biology differs from human biology. Clinical investigations focus on real patient outcomes and safety data, which is the case of our study. We did not include much preclinical investigation in the Discussion section, especially to avoid overinterpretation of preclinical evidence, while the components of Reparis and Cicatridina were used on human beings, but they were not investigated in such combination.

However, there is currently a lack of clinical trials investigating the effects of these natural products on wound healing [34]. Reparis is an innovative topical cream comprising 100% natural ingredients that uniquely combines modern science with the healing power of nature to promote wound healing and skin regeneration, with high efficacy across all areas of regeneration. The physicochemical properties of a topical formulation vehicle, such as creams, like Reparis and its excipients dictate active ingredient release, skin hydration, and tissue regeneration. Excipients modulate viscosity, pH, and thermodynamic activity, ensuring optimal drug partitioning into damaged skin layers to accelerate re-epithelialization, reduce inflammation, and enhance patient adherence. Rheological behavior ensures the formulation stays in place on a wound bed without excessively hindering the diffusion coefficient of active compounds, Lipophilic–hydrophilic balance (HLB) matching between excipients and active botanicals determines whether the drug molecules prefer leaving the vehicle to partition into the epidermis. Humectants like hyaluronic acid and natural emollients draw and retain water molecules, moisturizing at the lesion interface, which is critical for fibroblast proliferation and enzymatic autolytic debridement, establishing an optimal moist microenvironment that prevents scab-related micro-traumas during dressing changes, with a role in barrier restoration from external microbial invasion [38,39].

Cicatridina formulations rely on sodium hyaluronate as the core active ingredient. They utilize dual-action topical delivery systems—such as powder sprays with aluminum starch or lipid-based ointments with sweet almond oil—to optimize moisture control, provide structural tissue support, and accelerate re-epithelialization in superficial and deep wounds. Sweet almond oil and vitamin E acetate restore tissue elasticity, while tea tree and lemon essential oils or chlorhexidine offer antiseptic protection without damaging fragile granulation tissue. The use of an oil-in-water macro-emulsion (liquid paraffin, cetyl stearyl alcohol, and silicone oil) provides continuous occlusion and emollient therapy. Inaesthetic scar formation reduces patient compliance [40].

There is a large focus on plant extracts, delivered via modern hydrogels or topical creams. While thousands of preclinical studies exist, rigorous, high-level randomized controlled trials remain limited by small sample sizes and short follow-up windows [41]. *Centella asiatica* was investigated for its triterpenoid components (like asiaticoside and madecassoside) aimed at modulating collagen synthesis and preventing hypertrophic scars [42] *Aloe vera* was evaluated for its utility in accelerating re-epithelialization in acute and minor burn wounds through the polysaccharide-driven anti-inflammatory pathway [43]. But there is a concern regarding these clinical trials related to their heterogeneity, because of the variability in botanical extraction methods, dosing concentrations, and purity standards, their trial quality, due to a lack of rigorous blinding, small cohorts, and follow-ups under one year, and their delivery challenges, as low skin-barrier penetration and bioavailability require modern nanocarriers and hydrogels to translate bench success into verified clinical results [44,45].

## 4. Materials and Methods

The following is an observational retrospective study of collected data conducted in the Department of Obstetrics and Gynecology, County Hospital Hunedoara, between 26 February and 31 May 2026, evaluating specific measures that relate to cosmetic results for scars after a surgical procedure in our unit on 50 patients using Reparis twice a day compared to the control group, consisting of 50 patients using Cicatridine twice a day for 30 days, in a thin layer, beginning one day after surgery. Reparis (manufactured by Adexilis, Istanbul, Turkey) contains the following ingredients: hyaluronic acid, *Centella asiatica*, *Aloe vera*, *Hypericum perforatum*, *Camelia sinensis*, *Hippophae rhamnoides*, *Calendula officinalis*, *Melaleuca alternifolia*, vitamin E, and Dexpanthenol. Cicatridine (manufactured by Pharma-Derma SRL, Sala Bolognese, Italy) contains the following ingredients: Hyaluronic acid, chlorhexidine, almond oil, and silicon oil. The data collection took place between 1 January and 31 December 2025.

Reparis contains hyaluronic acid, provitamin B5 (dexpanthenol), and vitamin E with protective and hydration roles. In addition, it contains calming botanicals, such as *Centella asiatica*, *Aloe vera*, *Calendula*, and St. John’s wort, and nourishing oils, such as olive extract, sea buckthorn oil, green tea extract, tea tree oil, and squalane [13].

Reparis’s mechanism of action comprises an anti-inflammatory role to soothe redness, swelling, itching, and discomfort using tea tree oil, *Aloe vera*, and St. John’s wort. Collagen stimulation constitutes another mechanism that enhances tissue regeneration and angiogenesis via *Centella asiatica* and hyaluronic acid. Epithelialization promotes skin cell division and barrier recovery through *Calendula*, panthenol, and *Aloe vera*. Antioxidant defense is another mechanism that neutralizes free radicals with vitamin E and squalane to protect healing skin [13].

Cicatridine relies primarily on hyaluronic acid in combination with natural extracts such as *Aloe vera*, *Calendula*, and *Centella asiatica* to repair and moisturize damaged tissues. Its composition includes the core active ingredient that retains water, hyaluronic acid, used for hydration, in addition to natural herbal extracts such as *Centella asiatica*, *Calendula*, *Aloe vera*, and *Melaleuca* to soothe inflammation. Base excipients and oils such as sweet almond oil, liquid paraffin, or semi-synthetic glycerides are responsible for delivering a smooth, protective topical texture [14].

Cicatridina’s mechanism of action is as follows: tissue repair accelerates the re-epithelialization process for skin lesions; hydration aids in retaining local moisture to relieve dryness, irritation, and cracking in mucous membranes or skin; lastly, to provide a protective barrier, a film forms over wounds or irritated areas to shield them from external friction and pathogens [14].

Uniformity in polyherbal formulations such as Reparis or Cicatridina is managed through strict standardization protocols across every stage of production, from raw plant to final cream. Because the composition of natural plant materials varies with weather conditions, soil characteristics, and harvest timing, manufacturers rely on chemical profiling rather than plant weight alone.

Uniformity is achieved through the following measures: First, testing ensures that every batch contains a consistent percentage of specific active molecules (such as asiaticoside in *Centella asiatica* or polyphenols in green tea). Second, High-Performance Liquid Chromatography is employed to analyze each compound to create a chemical “fingerprint” that matches the master formula. Third, plants are grown under identical conditions, with herbs sourced from the same regions to prevent variations. Lastly, standardized extraction processes followed by rigorous testing are employed for standardization [15].

Inclusion criteria for this study were age ≥ 18 years, no evident cognitive impairment, provision of written informed consent, and the absence of skin lesions prior to scar formation. Exclusion criteria included age < 18 years, cognitive impairment, autoimmune diseases, dermatological diseases, insufficient understanding of the Romanian language, and noncompliance with planned follow-up procedures.

This study was approved by the Ethics Committee of County Hospital Hunedoara, 331057 Hunedoara, Romania (protocol code 3765/25 February 2026). Informed consent was obtained according to the principles of the Declaration of Helsinki.

Clinical patient characteristics included age, body mass index (BMI), parity, associated pathologies, laboratory analyses, complications during and after surgery, and length of hospitalization (in days); these data were obtained from patient medical records. Follow-up assessments were conducted at 1 week, 6 weeks, and 6 months after the procedure. All enrolled patients completed the POSAS (Patient and Observer Scar Assessment Scale), an internationally validated questionnaire used to evaluate both patient and observer assessments of scar appearance.

The five variables assessed comprise six items: scar-related pain, itchiness, color, stiffness, thickness, and irregularity. Each POSAS item is scored on a 10-point Likert scale, with 1 representing healthy skin and 10 the worst imaginable scar or sensation; these items are then summed to obtain a total score ranging from 6 to 60 for each scale. In addition to the POSAS score, both observer and patient provided their overall opinion on the appearance of the scar using a 10-point scale. The scale was administered by the attending physician to the patients at the first follow-up appointment, scheduled 7 days after surgery, and subsequently at 6 weeks and 6 months. The observers were blinded to the product used by each patient.

Each item score above 2 was considered pathological, with a total (sum of items) above 6 also considered pathological. We compared the results in both groups after 1 week, 6 weeks, and 6 months. We correlated the results with age, associated pathologies, intra- and post-interventional complications, laboratory tests, and hospital days.

The first outcome was acute repair through anti-inflammatory activity, reducing itching and pain.

The second, equally important outcome was chronic repair through tissue regeneration, collagen production, and angiogenesis stimulation.

### Statistical Analysis

Statistical analysis was performed using IBM SPSS Statistics for Windows, version 23.0, and Microsoft Excel. The chi-square test/Fisher’s exact test was used for categorical variables to determine associations or comparisons. Continuous parameters are expressed as median, range, minimum and maximum, and mean ± standard deviation (SD), while categorical variables are presented as numbers and percentages. Differences in continuous variables across categorical variables were tested using the Mann–Whitney U test. Spearman correlation was applied to determine the degree to which different parameters were related. A positive correlation coefficient (+) indicates that both variables tend to increase or decrease together, whereas a negative correlation coefficient (−) indicates that one variable increases as the other decreases. The independent samples *t*-test was used to compare the central tendency of the parameters. The significance level was set at 0.05, and *p* < 0.05 was considered statistically significant.

Because preliminary pilot data were not available in the literature, the sample size was set at 50 per group based on institutional feasibility. A post hoc sensitivity analysis demonstrated that this sample size provides 80% power at a two-sided α = 0.05 to detect a medium-to-large Cohen’s d effect size of 0.57.

## 5. Conclusions

Based on the findings of our observational study, natural compounds alone, such as Reparis, demonstrate promising effects on wound healing, with the most significant effect observed six months after treatment. Their effects may contribute to reductions in thickness, redness, and pain in the wound area. The effects of natural compounds could potentially be enhanced through the use of novel delivery systems. The therapeutic properties of traditional medical plants have been documented, but not always in controlled clinical trials. Much remains to be learned from traditional medicine.

## Figures and Tables

**Table 1:** Clinical characteristics.

Variables		Reparis N = 50	Cicatridine N = 50	*p*-Value
Age	Mean ± SD	27.50 ± 4.77	27.20 ± 4.78	0.75
BMI	Mean ± SD	24.48 ± 1.74	24.80 ± 2.17	0.42
Days of hospitalization	Mean ± SD	4.70 ± 1.31	4.72 ± 1.23	0.93
Parity	1	33 (66.0)	33 (66.0)	0.65
	2	13 (26.0)	10 (20.0)	
	3 and over	4 (8.0)	7 (14.0)	
Assoc. diseases	Autoimmune disease	1 (2.0)	0 (0.0)	0.55
	Diabetes mellitus	2 (4.0)	2 (4.0)	
	Hypothyroidism	0 (0.0)	1 (2.0)	
	Hyperthyroidism	1 (2.0)	0 (0.0)	
Lab. test	Anemia	7 (14.0)	8 (16.0)	0.95
	Glycemia	2 (4.0)	2 (4.0)	
	Le	4 (8.0)	5 (10.0)	
	N	37 (74.0)	35 (70.0)	
Complications during surgery	Hemorrhage	2 (4.0)	2 (4.0)	0.98
Complications after surgery	Infection	4 (8.0)	4 (8.0)	0.98

BMI, body mass index; Lab. test, laboratory test; Le, leucocytes; N, normal.

**Table 2:** POSAS at 1 week.

Groups		Pain	Itchiness	Color	Stiffness	Thickness	Irregularities	Total
Reparis	Median	1.00	1.00	1.00	1.00	1.00	1.00	6.00
	Range	5	6	6	6	7	7	37
	Minimum	1	1	1	1	1	1	6
	Maximum	6	7	7	7	8	8	43
Cicatridine	Median	1.00	1.00	1.00	1.00	1.00	1.00	6.00
	Range	0	2	1	1	1	2	3
	Minimum	1	1	1	1	1	1	6
	Maximum	1	3	2	2	2	3	9
*p*-value		0.042	0.17	0.0001	0.0001	0.0001	0.177	0.003

Data are expressed as medians and ranges. The Mann–Whitney U test was applied for comparisons between groups.

**Table 3:** POSAS after 6 weeks.

Groups		Pain	Itchiness	Color	Stiffness	Thickness	Irregularities	Total
Reparis	Median	1.00	1.00	1.00	1.00	1.00	1.00	6.00
	Range	0	2	4	3	3	1	12
	Minimum	1	1	1	1	1	1	6
	Maximum	1	3	5	4	4	2	18
Cicatridine	Median	1.00	1.00	1.50	1.00	1.00	1.00	7.00
	Range	0	1	4	3	3	1	10
	Minimum	1	1	1	1	1	1	6
	Maximum	1	2	5	4	4	2	16
*p*-value		0.98	0.43	0.36	0.45	0.98	0.64	0.49

Data are expressed as medians and ranges. The Mann–Whitney U test was applied for comparisons between groups.

**Table 4:** POSAS after 6 months.

Groups		Pain	Itchiness	Color	Stiffness	Thickness	Irregularities	Total
Reparis	Median	1.00	1.00	1.00	1.00	1.00	1.00	6.00
	Range	0	1	3	3	3	0	8
	Minimum	1	1	1	1	1	1	6
	Maximum	1	2	4	4	4	1	14
Cicatridine	Median	1.00	1.00	1.50	1.00	2.00	1.00	9.00
	Range	0	1	6	5	6	2	19
	Minimum	1	1	1	1	1	1	6
	Maximum	1	2	7	6	7	3	25
*p*-value		0.98	0.31	0.001	0.0001	0.0001	0.0001	0.0001

Data are expressed as medians and ranges. The Mann–Whitney U test was applied for comparisons between groups.

**Table 5:** Correlations for Reparis.

Reparis		Total/1 w	Total/6 w	Total/6 m
Age	*rho* Spearman	0.159	0.313	0.164
	*p*-value	0.271	0.027	0.255
BMI	*rho* Spearman	0.009	0.039	0.298
	*p*-value	0.951	0.787	0.056
Days of hosp.	*rho* Spearman	0.444 **	0.576 **	0.298
	*p*-value	0.001	0.0001	0.036

** Correlation is significant at the 0.01 level (2-tailed).

**Table 6:** Correlations for Cicatridine.

Cicatridine		Total/1 w	Total/6 w	Total/6 m
Age	*rho* Spearman	0.433 **	0.422 **	0.444 **
	*p*-value	0.002	0.002	0.001
BMI	*rho* Spearman	0.406 **	0.554 **	0.519 **
	*p*-value	0.003	0.0001	0.0001
Days of hosp.	*rho* Spearman	0.659 **	0.679 **	0.708 **
	*p*-value	0.0001	0.0001	0.0001

** Correlation is significant at the 0.01 level (2-tailed).

## Data Availability

The original contributions presented in this study are included in the article. Further inquiries can be directed to the corresponding author.

## Abbreviations

The following abbreviations are used in this manuscript:

C.	asiatica *Centella asiatica*
FGF	Fibroblast Growth Factor
VEGF	Vascular Endothelial Growth Factor
IL-1β	Interleukin-1β
IL-6	Interleukin-6
TNFα	Tumor Necrosis Factor α
